# Maturity of defense mechanisms as a predictor of adherence to treatment in patients with cardiovascular diseases

**DOI:** 10.1192/j.eurpsy.2025.1228

**Published:** 2025-08-26

**Authors:** E. V. Deshchenko, E. I. Pervichko, E. V. Akatova

**Affiliations:** 1 Lomonosov Moscow State University; 2 Russian University of Medicine of the Ministry of Health of Russia, Moscow, Russian Federation

## Abstract

**Introduction:**

Promoting adherence to treatment is essential for increasing the effectiveness of therapy of the cardiovascular diseases. The role of defense mechanisms and frustration reactions of the cardiac patients in their adherence to treatment was not studied previously.

**Objectives:**

The aim of the research was to study the relationship between defense maturity and adherence to treatment in patients with cardiovascular diseases.

**Methods:**

To measure the adherence to treatment the Questionnaire for Comprehensive Assessment of Treatment Adherence was used (Nikolayev *et al*. Clinical Pharmacology and Therapy 2018, 1 74-78). Defense mechanisms were assessed using the Defense Mechanisms Rating Scales (DMRS-SR-30; Di Giuseppe *et al*. Front. Psychiatry 2020, 11:870). The Picture Frustration Test was used to assess patients’ frustration reaction types (Rosenzweig. Journal of Personality 1945, 14 3-23). Structural equation modeling (path analysis method) was used for data analysis. The study was conducted from December 2022 to April 2023. The sample consisted of 42 male patients hospitalised with multiple cardiac pathology, whose average age was 49.40±7.7.

**Results:**

The majority of the patients in our sample demonstrated middle level of the adherence to treatment, with mean score being 61.17±18.53%. Twelve (30%) participants were defined as low-adherent and nine (22.5%) were defined as high-adherent. Assessment of frustration reactions showed that the adherence to treatment is positively connected with intropunitive and need-persistent reactions (r=0.428, p=0.013; r=0.459, p=0.007) and negatively connected with extrapunitive reactions (r=-0.409, p=0.004). What concerns defense mechanisms, the maturity of defenses appeared to be positively connected with the adherence to treatment (r=0,388, p=0,021). Using path analysis, we found only one theoretical model to be representative of the empiric data. The model constructed reveals indirect effect of the defense maturity on the adherence to treatment, mediated by the type of frustration reaction. The paths are positive, significant and equal 0.242 (χ2=1.871, p=0.171, CFI=0.947, RMSEA=0.176). Patients that use more mature defenses are more likely to give need-persistent reactions to frustration, hence, they have better adherence to treatment.

**Conclusions:**

Thus, the results of our study demonstrate that defense maturity can be used as a predictor of the adherence to treatment in patients with cardiovascular diseases due to its role in determining the type of frustration reactions. More mature defenses allow patients to be more flexible in their reaction to the illness and its limitations that leads to higher adherence to treatment.

**Disclosure of Interest:**

None Declared

